# Home

**Published:** 1877-10

**Authors:** 


Have the children do some little things, daily, for your personal comfort; let them see that it gives you pleasure, and that you depend on them for the service. This will attach them to you more strongly; and if they feel responsibility, even in matters of themselves trivial, and are sure of your sympathy, their affections and joys will cluster around the home hearth.

"Children like to be useful—it makes them happy. So give them work-time as well as play-time. But in any case, and in all cases give them sympathy. Express love for them."

---

More than building showy mansion,
More than dress and fine array,
More than domes or lofty steeples,
More than station, power, and sway,
Make your home both neat and tasteful,
Bright and pleasant, always fair,
Where each heart shall rest contented,
Grateful for each beauty there.

More than lofty, swelling titles,
More than fashion's luring glare,
More than mammon's gilded honors,
More than thought can well compare,
See that home is made attractive
By surroundings pure and bright,
Trees arranged with taste and order,
Flowers, with all their sweet delight.

Seek to make your home most lovely:
Let it be a smiling spot,
Where, in sweet contentment resting,
Care and sorrow are forgot;
Where the flowers and trees are waving
Birds will sing their sweetest songs,
Where the purest thoughts will linger,
Confidence and love belongs.

Make your home a little Eden;
Imitate her smiling bowers;
Let a neat and simple cottage
Stand among bright trees and flowers.

There, what fragrance and what brightness
Will each blooming rose display,
Here a simple vine-clad arbor
Brightens through each summer day.

There each heart will rest contented,
Seldom wishing far to roam,
Or, if roaming, still will cherish
Mem'ries of that pleasant home;
Such a home makes man the better,
Pure and lasting its control;
Home with pure and bright surroundings
Leaves its impress on the soul. ANON.

